# Our “energy-Ca^2+^ signaling deficits” hypothesis and its explanatory potential for key features of Alzheimer’s disease

**DOI:** 10.3389/fnagi.2014.00329

**Published:** 2014-12-03

**Authors:** Ming Chen, Huey T. Nguyen

**Affiliations:** ^1^Aging Research Laboratory, Research and Development Service, Bay Pines Veterans Affairs Healthcare SystemBay Pines, FL, USA; ^2^Department of Molecular Pharmacology and Physiology, University of South FloridaTampa, FL, USA

**Keywords:** Alzheimer’s disease, energy, calcium, amyloid, tau

## Abstract

Sporadic Alzheimer’s disease (sAD) has not been explained by any current theories, so new hypotheses are urgently needed. We proposed that “energy and Ca^2+^ signaling deficits” are perhaps the earliest modifiable defects in brain aging underlying memory decline and tau deposits (by means of inactivating Ca^2+^-dependent protease calpain). Consistent with this hypothesis, we now notice that at least eight other known calpain substrates have also been reported to accumulate in aging and AD. Thus, protein accumulation or aggregation is not a “pathogenic” event, but occurs naturally and selectively to a peculiar family of proteins, and is best explained by calpain inactivation. Why are only calpain substrates accumulated and how can they stay for decades in the brain without being attacked by many other non-specific proteases there? We believe that these long-lasting puzzles can be explained by calpain’s unique properties, especially its unusual specificity and exclusivity in substrate recognition, which can protect the substrates from other proteases’ attacks after calpain inactivation. Interestingly, our model, in essence, may also explain tau phosphorylation and the formation of amyloid plaques. Our studies suggest that α-secretase is an energy-/Ca^2+^-dual dependent protease and is also the primary determinant for Aβ levels. Therefore, β- and γ-secretases can only play secondary roles and, by biological laws, they are unlikely to be “positively identified”. This study thus raises serious questions for policymakers and researchers and these questions may help explain why sAD can remain an enigma today.

## A CONCEPTUAL CRISIS IN ALZHEIMER’S DISEASE STUDY

The study of Alzheimer’s disease (AD) is in crisis today. Although progress has been made in understanding early-onset AD (a rare disease mostly caused by gene mutations), late-onset sporadic AD (LOAD or sAD), which affects the vast majority of AD victims and is the main threat to modern society, has remained an enigma after nearly 40 years of intensive studies (Holtzman et al., 2012). This problem has motivated policymakers in Congress and funding agencies to call for increased funding and innovative researches.

But we think it is more important to understand why the crisis has happened. To this end, we have undertaken an independent analysis of the current issues and realized that the official definition for sAD, a *senile disorder*, as a “discrete disease” by the National Institute on Aging (NIA) may be the root problem. This definition has ignored the fundamental differences between senile disorders and discrete diseases in origin, study paradigm and intervention approach. Our study emphasizes that sAD, a devastating disease in social impact, but is also a *normality* in its biological nature (like hearing loss and heart failure at advanced age). This should be a new conceptual basis to understand the disorder (Chen and Fernandez, 2000, 2001a; Chen et al., 2011a,b).

As the NIA definition has overlooked the unique features of sAD, it has consequently confined the studies to the prominent “pathological” lesions (e.g., plaques and tangles) and presumed “abnormal” pathways (e.g., gene mutations or Ca^2+^ overactivation). Such studies, though well-intentioned and highly productive, may never explain the basic features of sAD (e.g., why it is a result of demographic changes and why it increases exponentially with age). As such, these studies, though being praised enthusiastically by the AD study field and mass media, have never been accepted by the general medical community (e.g., NIH independent committee consensus statements 2002 and 2010; see NIA website).

This may be why, after so many years with over 130,000 research papers published and many of which are in prestigious journals, sAD can remain a conceptual enigma accompanied by repetitive failures in clinical trials, a crisis unseen in medical history. Thus, a high priority today is to synthesize current data into novel hypotheses that can explain sAD features better than the existing ones, thereby guiding future studies in a new direction.

## OUR HYPOTHESIS FOR THE ORIGINS OF sAD

**FIGURE 1:**
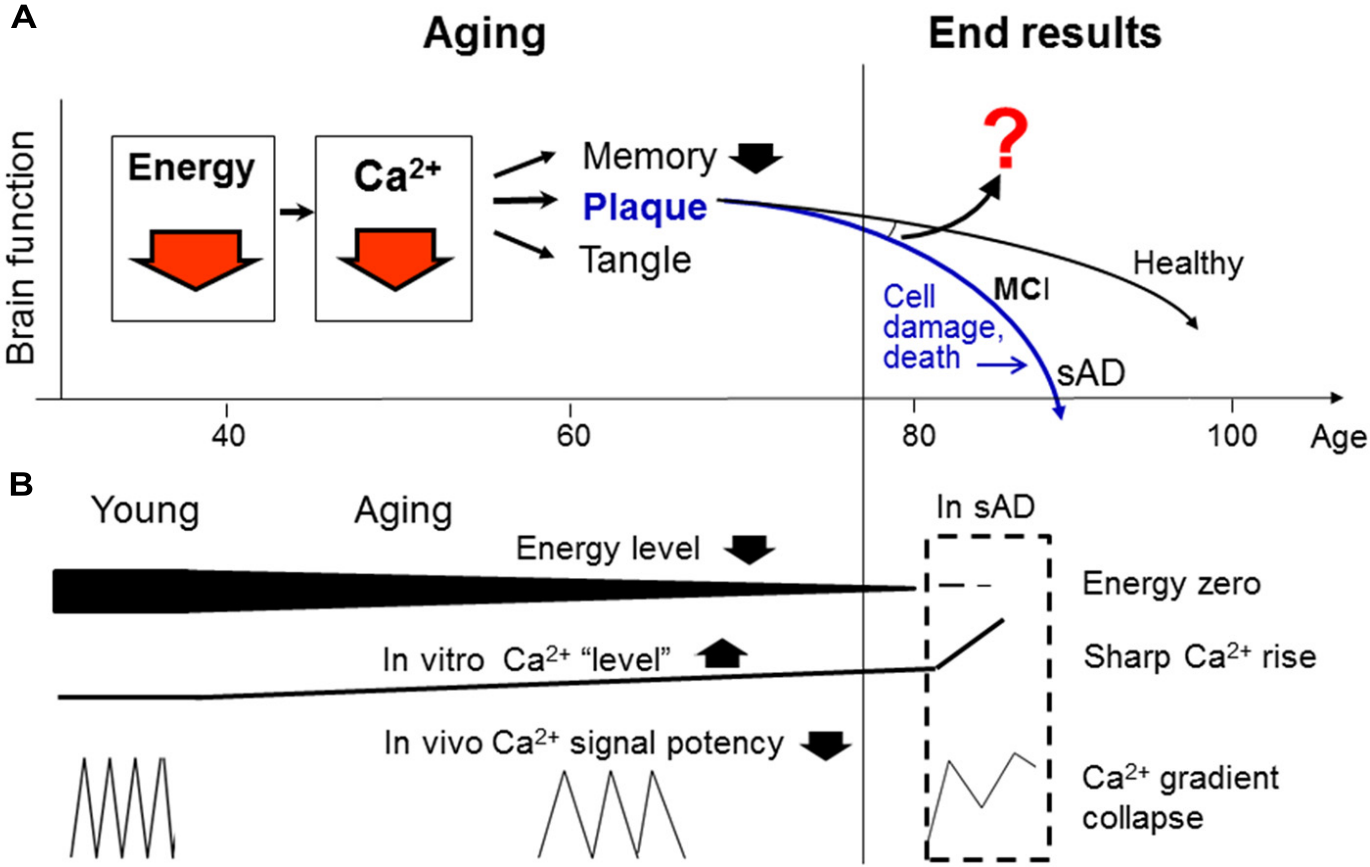
**An integrative model for the “natural history” of sporadic Alzheimer’s disease (sAD). (A)** Proposes that energy deficiency is the earliest modifiable defect in brain aging, which causes, among other things, a Ca^2+^ signaling deficit underlying inefficient memory and the formation of plaques and tangles (by means of inactivating Ca^2+^-dependent proteases and phosphatases; not shown, see text below). **(B)** Explains why energy deficit decreases Ca^2+^ signal potency (by reducing Ca^2+^ wave frequency), and why this change can manifest as slow rising Ca^2+^ “levels” during aging and sharp Ca^2+^ rises in cell death. As the aging process continues into advanced stage, it will diverge into various end results in the elderly population ranging from healthy brain to MCI or sAD. Thus, the reasons for the divergence should be a supreme question to answer (question mark; see text below). Despite the remaining questions, our model points to energy and Ca^2+^ deficits as two ideal points of entry for early sAD intervention (red arrows), whereas targeting the prominent lesions themselves (plaques and tangles) or numerous cell death-associated impairments will not have any therapeutic values. The current “amyloid hypothesis” (blue color) is also shown for comparison.

In this context, we and a growing number of other investigators have started to think that sAD should be understood from a new perspective, i.e., aging (Chen, 1998; Swerdlow, 2007; Yankner et al., 2008; Castellani et al., 2009; Herrup, 2010; Sperling et al., 2011; Korczyn, 2012). From this ground, we have proposed a new hypothesis for the natural history of sAD (**Figure 1**). This hypothesis is unique in that it divides the widely called “AD process” into two distinctive stages: normal aging and cell-death stages. Emphasizing the normal aging stage is because no sAD case occurs without having passed through a long aging process. Also, unlike other models that focus on the prominent “pathological” lesions or cellular impairments in the latter stage (Holtzman et al., 2012), our hypothesis considers the former stage as the primary study focus because we believe that similar to other *senile disorders,* sAD is initiated from *invisible* and *normal* changes during *natural* aging, and only such changes are the reasonable drug targets for its intervention (Chen et al., 2011b).

But, among the myriad changes in the aging brain, which one(s) should receive our primary attention? In this regard, we (Chen and Fernandez, 2001a) and others (Beal, 1998; Lannert and Hoyer, 1998; McDaniel et al., 2003; Höglinger et al., 2005; Moreira et al., 2010; Swerdlow, 2012) have suggested that energy/mitochondrial dysfunction is perhaps the *earliest modifiable* defect. Indeed, from the viewpoint of bioenergetics, life itself is merely a process of energy generation and consumption, and free energy is the ultimate driving force for physiological activities, especially neurotransmission and memory (Chen and Fernandez, 2000; Chen et al., 2011b; **Figure 1**). We also believe that energy deficit in most elderly is a naturally-occurring event, one that is not triggered by any “pathogenic/erroneous” factors, except for the passage of time.

By what mechanism can energy affect memory? We reasoned that because Ca^2+^ is a central regulator in neurotransmission and Ca^2+^ signaling is highly energy-dependent, energy deficit must cause memory inefficiency primarily by means of inactivating Ca^2+^ signaling, amongst its many consequences in the body (e. g., free radicals, bone loss, etc.) (Chen et al., 2011b). This will happen with concomitant formations of plaques and tangles (by means of inactivating Ca^2+^-dependent proteases such as calpain; tau is a known calpain substrate; plaques may be formed by a similar mechanism, see below; Chen and Fernandez, 2001b; Chen et al., 2011b; **Figure 1**). It thus appears that the proposed energy and Ca^2+^ deficits can uniformly explain the three diagnostic markers of sAD: memory decline, plaques and tangles.

The model also suggests that additional factors (question mark; **Figure 1**) are required to diverge the aging process into various end results in the elderly (see below). In contrast, the current “amyloid hypothesis” (blue color) does not explain two basic questions: (i) what has caused the plaque formation; and (ii) why many elderly remain healthy despite the presence of plaques and tangles.

### CONTROVERSIES IN Ca^2+^ CHANGES IN AGING

Interestingly, while energy depletion is a well-accepted concept, the proposed “Ca^2+^ deficit”, a logical consequence of energy deficit, has nevertheless become a primary point of controversy, because it confronts the current “Ca^2+^ overload/activation” hypothesis (Khachaturian, 1994), a doctrine that has been taken as granted by numerous studies (e.g., Manya et al., 2002; Sloane et al., 2003; Ferreira, 2012). As we have discussed extensively (Chen and Fernandez, 1999, 2001a,b; Chen et al., 2011b), however, the latter hypothesis is questionable because it: (i) derives from the flawed “disease” definition of sAD and thus rests on a presumptive “abnormal” pathway as its “cause”; (ii) defies the commonsense knowledge (e.g., how can the energy-dependent Ca^2+^ signaling be activated in the energy-depleted aging process?); and (iii) has not been supported by consistent clinical data after many trials, but is challenged by at least three studies reporting that calcium antagonists exhibit negative effects on cognition in the elderly (Heckbert et al., 1997; Maxwell et al., 1999; Wagner et al., 2012).

In contrast to this hypothesis, our model is in line with the clinical studies (Heckbert et al., 1997; Maxwell et al., 1999; Ritchie et al., 2007; Newhouse et al., 2012; Wagner et al., 2012) and also with several important reports showing that a number of Ca^2+^-dependent enzymes/factors are inactivated during aging (Baudry et al., 1986; Guttmann et al., 1997; Gao et al., 1998; Pahlavani and Vargas, 1999; Agbas et al., 2005; Moriguchi et al., 2006; Pascale et al., 2007; Rice et al., 2014; Sun et al., 2014). Most importantly, the model can logically explain several basic features of sAD (see above).

In support of this model, our experimental studies have recently demonstrated that the Ca^2+^ signaling efficacy and calpain activity are both *decreased* in old human fibroblasts, despite the elevated steady-state Ca^2+^ levels there. While much more studies are required for definitively resolving this issue, our findings suggest that higher static Ca^2+^ “levels” in aged cells result from energy depletion and lead to lower Ca^2+^ signaling potency. This intriguing phenomenon may happen because the frequency and amplitude of the Ca^2+^ waves *in vivo* may reduce by aging, but this change can manifest as a “Ca^2+^ overstay” or elevated steady-state “levels” *in vitro* (**Figure 1B**). For this reason, the rational approach to reduce the observed “higher Ca^2+^ levels” in the aging brain cells should make use of energy metabolism stimulators and Ca^2+^ agonists, not antagonists as currently believed (Nguyen et al., 2013).

Furthermore, we have also found that Ca^2+^/calpain is indeed sharply activated in the dying cells (Nguyen et al., 2013), suggesting that Ca^2+^/calpain undergoes a “bi-phasic” change in the “sAD process”: first inactivated during aging, then dramatically activated at the cell-death stage, as observed in the AD brain (Saito et al., 1993). The latter change, however, may not have any therapeutic values (**Figures 1A,B**).

## SELECTIVE ACCUMULATION OF CALPAIN SUBSTRATES IN AGING

Is this hypothesis reasonable? Despite the supportive data, it must be kept in mind that the dynamic Ca^2+^ changes in the living brain have not been directly determined by current technologies, so any hypotheses about them should be scrutinized with caution. One way to do this is by examining their corollaries. An important corollary of our model is that if the Ca^2+^/calpain deficit causes the deposition of tau, then it should also cause the concomitant depositions of many other known calpain substrates. Is this the case? This would be a key test for our model.

To this end we undertook a review of literature and found that, amazingly, at least eight other proteins have also been reported to accumulate or aggregate in aging and AD. They are: spectrin, crystalline (in cataracts), filamin, myosin, MAP2, calcineurin, tubulin, and neurofilament proteins (Johnson et al., 1991; Ashford et al., 1998; Biswas et al., 2004; Agbas et al., 2005; Puig et al., 2005; Feuillette et al., 2010; Yan et al., 2012), which are all well-known calpain substrates (Goll et al., 2003; a comprehensive review). These accumulated proteins, if considered together, would have profound implications for the origins of sAD and for our proposal, as it suggests that protein accumulation during aging is not a “pathogenic” event, but occurs naturally and preferentially to a peculiar protein family.

But discrepancies exist. For example, proteins other than calpain substrates have also been reported to accumulate in the AD brain (Griffin et al., 1989). However, it appears that only calpain substrates have been consistently reported by various laboratories to progressively accumulate from early aging and throughout the aging and AD process.

Also, because the accumulated proteins are the breakdown products of well-known calpain substrates, they have been widely called “calpain-cleaved products,” thereby suggesting “calpain overactivation” (Biswas et al., 2004; Feuillette et al., 2010; Yan et al., 2012). This view, however, needs to be revisited for two reasons. First, it implies “the more protease cleavage, the more substrate accumulation,” a scheme that sounds illogical. Second, it has overlooked the fact that calpain is essential for physiological activities such as cell division and growth. As such it should reach maximum activity in the *young*, but none of its products is accumulated there. So it is reasonable to believe that *genuine* calpain-cleaved products will not accumulate even at their peak levels. Therefore, the accumulated fragments in aging and AD brains should be the *alternatively cleaved* products by other proteases after calpain inactivation. Sequencing of these products and comparing them with genuine calpain-cleaved ones will confirm this view.

Yet, it is also widely believed today that the accumulated proteins may not result from insufficient proteolysis, but from *abnormal* aggregation or phosphorylation by *abnormally activated* protein kinases (e.g., tau; Chung, 2009; Bolshette et al., 2014). We have reservations for these views because they have not explained why *abnormal* events can happen in *normal* early aging during which the proteins start to aggregate, and especially why the aggregation occurs selectively to calpain substrates.

The issues are still open for debate together with many other alternative views [e.g., Ca^2+^/calpain is unchanged in normal aging (Saito et al., 1993; Ito et al., 1994); tau is a caspase substrate (Fasulo et al., 2000); or aggregation is due to protein misfolding or autophagy (Nixon, 2013; Bolshette et al., 2014)]. It must be noted, however, that comparing with these other competing models, our hypothesis may offer the simplest and coherent explanation for the accumulation of several calpain substrates by a uniform mechanism.

## WHY ARE CALPAIN SUBSTRATES SELECTIVELY ACCUMULATED?

Now, why are calpain substrates selectively accumulated, how can they stay for decades in the brain without being attacked by many non-specific proteases there? And why does this not happen to other proteins? These long-standing puzzles need to be explained, but notably, they may not be easily explained by current models such as protein misfolding or autophagy (for their lack of selectivity). Thus we think that a clue should lie in the unique properties of calpain.

It is well-known that unlike most proteases that act non-specifically or randomly, calpain only makes limited cleavages on certain enzymes/proteins at designated sites and activates them for physiological functions. A well-known example is the cleavage of PKC by calpain to activate the PKC-related signal pathways (Kishimoto et al., 1989). Notably, such a cleavage *in vivo* must be highly specific, perhaps also *exclusive*, because if PKC is cleaved by any other proteases at the same site, disruption of signal transductions would result. We think that this could be a critical but as-yet-undocumented feature of the calpain-substrate interaction.

How can calpain ensure such a specificity and exclusivity in substrate recognition, given that it does not have a strong sequence recognition preference on its substrates (Friedrich and Bozóky, 2005)? For this puzzle, it has been speculated that calpain may recognize a unique and as-yet-unknown configuration in the secondary/tertiary structures of its substrates (Carafoli and Molinari, 1998; Cuerrier et al., 2005).

## A “LIGAND-RECEPTOR” MODEL FOR CALPAIN-SUBSTRATE RECOGNITION

**FIGURE 2:**
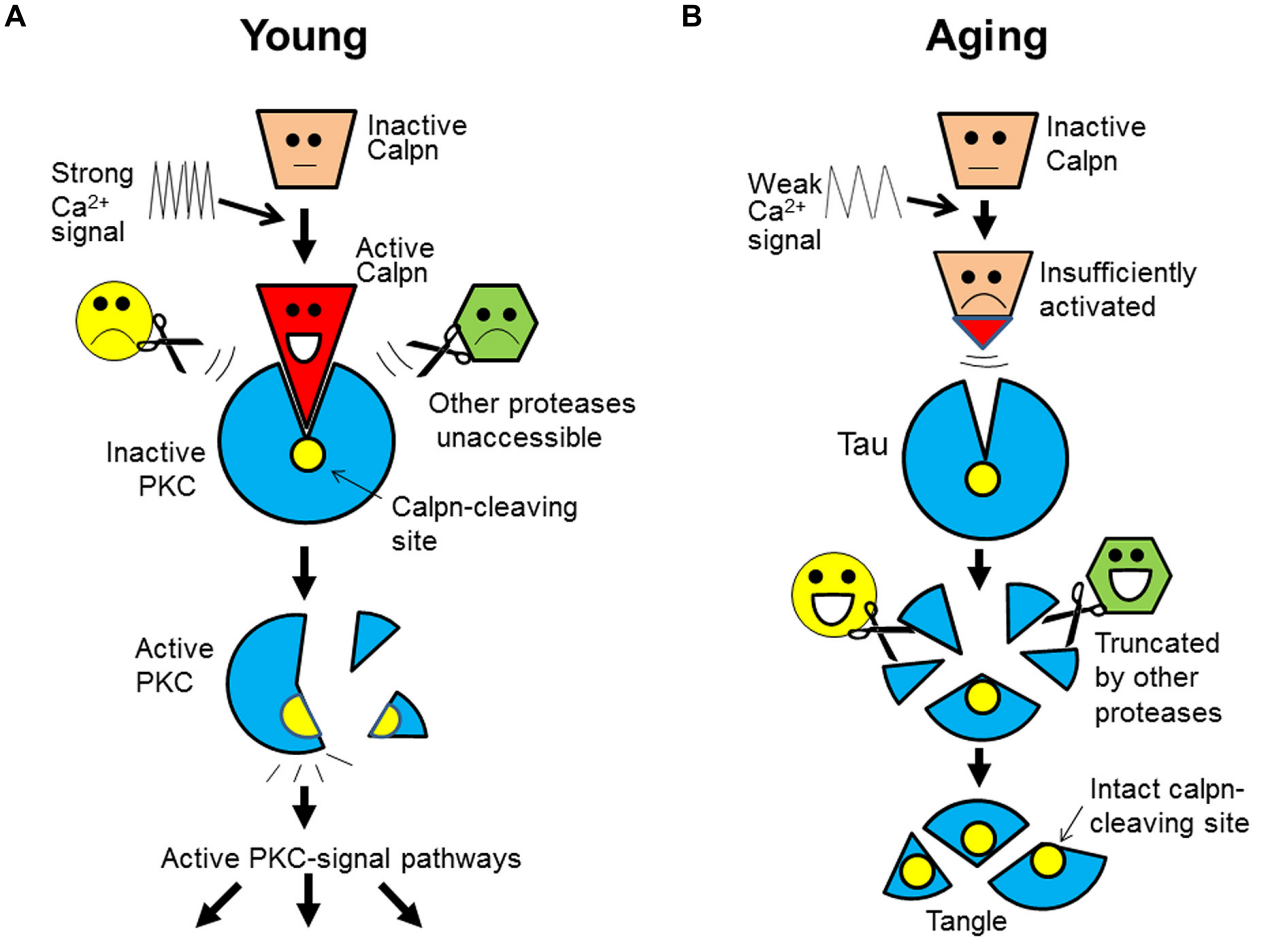
**A “ligand-receptor” model for calpain-substrate interaction and selective accumulation of calpain substrates in brain aging. (A)** In the young, calpain substrate (e.g., PKC) assumes a “receptor-like” configuration that is exclusively accessible by active calpain (as ligand). This unique relationship safeguards the specificity and exclusivity of their interaction, which allows a faithful cleavage/activation of PKC in the signal transduction free of interferences by any other proteases. **(B)** During aging, however, calpain is insufficiently activated as a result of energy and Ca^2+^ signaling deficits (slowed Ca^2+^ waves). So, calpain substrates (e.g., tau) will remain intact because other proteases will not cleave it at the calpain-leaving sites. Over time, tau will be truncated by other proteases at other sites, but its core fragments encompassing the calpain-cleaving sites will be spared and deposited (as tangles). Calpn, calpain.

Based on the current information, we further notice that the specificity and exclusivity of calpain-substrate interaction is quite similar to that of *ligand-receptor* (or antigen–antibody). From this new angle, we now propose that calpain substrates *in vivo* may assume an unusual, receptor-like configuration that is *exclusively* accessible by calpain. Upon activation, calpain may change its conformation in such a way that allows itself to fit into the substrate. Such a key-and-lock fitting would safeguard the specificity and exclusivity of their interactions free of interferences by any other proteases (**Figure 2A**; using PKC as an example).

Furthermore, of great interest is that this model may also explain the selective accumulation of calpain substrates. The receptor-like configuration of the substrates implies that after calpain inactivation, its substrates (such as tau) would not be attacked by any other proteases at the same sites. For this reason, the substrates would accumulate and then be truncated by other proteases at other sites, but their core fragments encompassing the calpain-cleaving sites (perhaps the domains recognized by calpain) would be spared and deposited over time, as undigested protein remnants (e.g., tangles; **Figure 2B**).

This model can explain: why only calpain substrates are protease-resistant; why the deposited tau is truncated at both ends (Guillozet-Bongaarts et al., 2005) and contains the predicted calpain-cleaving sites (Yang and Ksiezak-Reding, 1995); why the accumulated breakdown products from genuine calpain substrates are not genuine calpain-cleaved products, but are *alternatively cleaved* ones (see above); and more importantly, why protein accumulation is invariably *accompanying* memory decline during aging (both events are regulated by Ca^2+^), but targeting the deposited proteins themselves has not delayed the progression of dementia in clinical trials (protein deposits are secondary to the initial defects; **Figure 1**).

Can the proposed “ligand-receptor” model be experimentally tested? The model predicts that (i) distinct calpain substrates may share a unique secondary/tertiary configuration complementary to that of calpain in their crystal structures; (ii) deleting or mutating key amino acids in the unique configuration will abolish the substrates’ sensitivity to calpain; and (iii) such a configuration may not exist in other proteins. These corollaries may aid experimental validation of the model.

## WHY IS TAU PHOSPHORYLATED?

Very interestingly yet, our “ligand-receptor” model in its essence may also explain tau phosphorylation from a new perspective. Recall that there have been two basic scenarios for tau phosphorylation: overactivation of protein kinases, or inactivation of protein phosphatases (Trojanowski and Lee, 1995). Although the former is a current favor, questions remain as to whether kinases can be “activated” in normal aging where most enzymes loss activities (see above and below).

So the latter scenario is more reasonable. But, which phosphatase among numerous in the brain should we focus on? This key question has long limited the progress of the study area with a standing dilemma: if any phosphatase is inactivated, the loss will be compensated by many other non-specific phosphatases, so how can tau stay phosphorylated for decades in the brain?

These and other considerations thus lead us to a unique protein phosphatase, calcineurin, a Ca^2+^-dependent enzyme, of which tau is a well-known substrate (Kayyali et al., 1997; Yu et al., 2006; Karch et al., 2013). As a *regulated* enzyme, its substrate recognition should also be specific and exclusive to safeguard the integrity of signal transduction (similar to calpain-substrate interaction; **Figure 2**). Thus, as a physiological substrate of both calpain and calcineurin, tau may also assume a receptor-like configuration that is exclusively accessible by calcineurin. This would prevent tau from dephosphorylation by any other phosphatases at the same sites after calcineurin inactivation.

Thus, as a result of Ca^2+^ signal down-regulation during aging, tau would deposit and stay phosphorylated in the brain (Chen and Fernandez, 2001b). The two natural events can reinforce each other to render tangles a prominent feature of sAD, and thus there is no need to assume an *abnormal* mechanism for it.

Meanwhile, if other phosphatases are believed to be responsible for tau overphosphorylation, then it needs to explain why their activity loss is not compensated by many non-specific phosphatases in the aging brain.

## HOW ARE AMYLOID PLAQUES FORMED?

While our model may explain tangles and other protein deposits, it also raises a cardinal question: is amyloid-β precursor protein (APP) also a calpain substrate? This touches a sensitive issue because if it is, then calpain would mediate its normal processing, or as α-secretase as we suggested but controversial (Chen et al., 2000; Chen and Fernandez, 2004, 2005). But if it is not, then plaques would remain unexplained, because a key prediction of our model is that only the substrates of *regulated* proteases will deposit during natural aging (**Figure 2**).

So, the cardinal question becomes: is α-secretase a signal transduction-regulated protease? This point should be clear by now because numerous studies have shown that α-secretase is sensitively regulated by many signal transduction pathways, most notably glutamatergic, cholinergic, ERK/MAPK-, and PKC-related pathways (Buxbaum et al., 1990; Efthimiopoulos et al., 1996; Slack et al., 1997; Jolly-Tornetta et al., 1998).

But, while these elegant studies point to α-secretase as a regulated protease, they have not suggested a reasonable target for sAD intervention, since it is difficult to target all those pathways in practice. So a unifying model is needed, which should make fewest assumptions in order to parsimoniously and coherently explain most reports, thereby providing a simple drug target for intervention.

In this context, we proposed several years ago that α-secretase is a Ca^2+^-dependent protease, since Ca^2+^ appears to be a common denominator for those pathways (Chen, 1997). This view, though controversial, has since been strongly corroborated by an array of more recent reports, which have directly or indirectly linked α-secretase activity to Ca^2+^ (Mathews et al., 2002; Adlerz et al., 2007; Marcello et al., 2007; Dreses-Werringloer et al., 2008; Hoey et al., 2009; Vingtdeux et al., 2010; Suh et al., 2011; Zeiger et al., 2013; Kyratzi and Efthimiopoulos, 2014).

We thus believe that the concept of a Ca^2+^-dependent α-secretase is established and this offers a practical means for intervention even though its identity remains elusive. It is also noteworthy that because Ca^2+^ is the most sensitive and most exquisite regulator in the body, this can explain why APP α-processing is also sensitive to innumerable other elements such as cholesterol, cytokins, sirtuin, nardilysin, tetraspanin, and TIMP3 (Lichtenthaler, 2011; review).

Contrary to our view, however, an influential report has suggested that Aβ genesis, which changes in opposite direction to α-secretase, is a “Ca^2+^-dependent process” (Querfurth and Selkoe, 1994). But, it should be pointed out that physiological Ca^2+^-dependent processes [neurotransmission, cell growth, muscle contraction, etc. (Berridge et al., 1998)] are all reducing their activities during aging. Thus it is highly unlikely that Aβ genesis, which increases with aging, would be a *physiological* Ca^2+^-dependent process, even though it can be elevated by a Ca^2+^ stimulator at certain concentrations in the test tube.

Additional to its Ca^2+^-dependent feature, we and others have shown that α-secretase is also an energy-dependent protease, because its activity fluctuates intimately with cellular energy levels (Gasparini et al., 1997; Hoyer et al., 2005; Sawmiller et al., 2012). This view, at first glance, seems incompatible with the knowledge that proteolysis *in vivo* is generally a spontaneous event, and it is protein synthesis that is energy-dependent. So how can α-secretase be energy-driven? This question can be explained by the fact that Ca^2+^ signaling *per se* is highly energy-dependent (Walsh et al., 2009). This means that energy and Ca^2+^ signaling *in vivo* will undergo changes hand-in-hand. Moreover, APP α-processing, as part of protein secretions essential for cell maintenance and growth, will not occur spontaneously but must be controlled by energy supply and physiological demand. The energy-/Ca^2+^-dual dependent features of α-secretase provide a mechanism for such a control and, thus, they should be the “signature traits” of the enzyme to aid its identification.

## ADDITIONAL EVIDENCE FOR α-SECRETASE BEING ENERGY-/Ca^2+^-DUAL DEPENDENT

The proposed “signature traits” for α-secretase have touched a central yet most controversial issue in the sAD study: the mechanism of the plaque formation. So we further examined them by measuring α-secretase activity in response to five agents in a comprehensive way. The agents are ATP, nicotine, glutamate, epidermal growth factor (EGF), and phorbol 12,13-dibutylester (PDBu), which represent the five most important signal pathways regulating α-secretase: energy-, cholingeric, glutamatergic, ERK/MAPK-, and PKC-related pathways, respectively, (Buxbaum et al., 1990; Efthimiopoulos et al., 1996; Slack et al., 1997; Jolly-Tornetta et al., 1998; Sawmiller et al., 2012).

**FIGURE 3:**
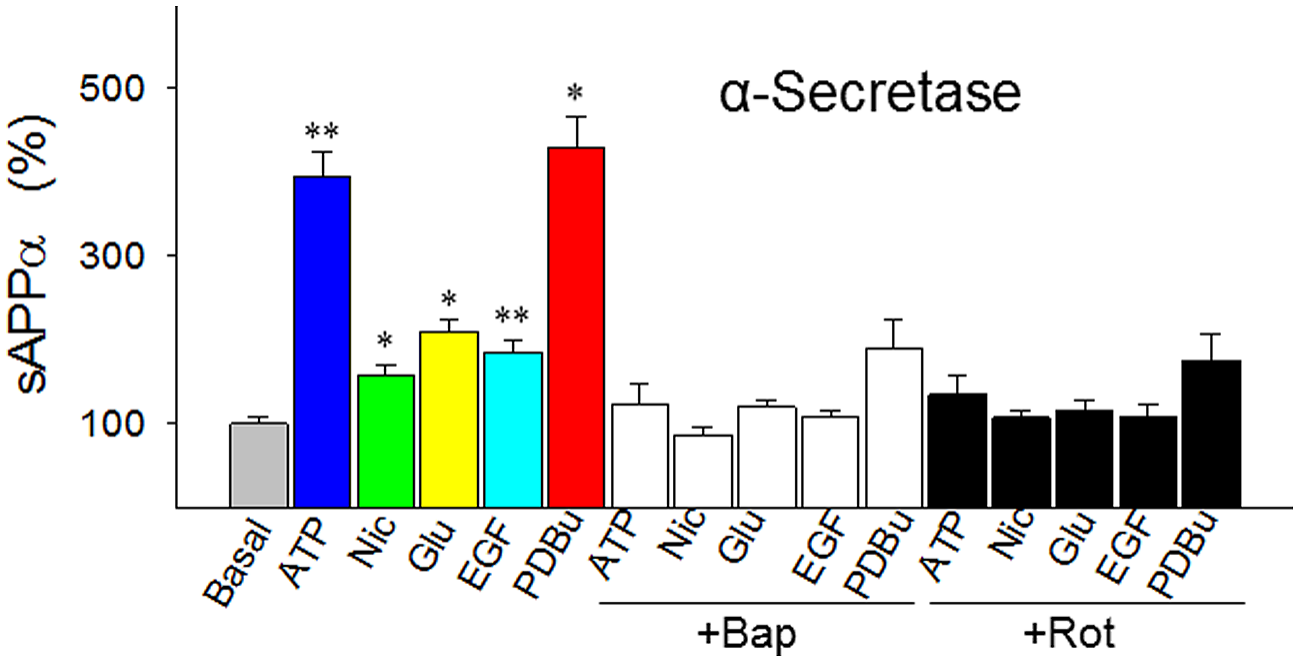
**Responses of α-secretase activity (measured as sAPPα levels) to five agents that represent the five best known metabolic/signal pathways regulating α-secretase (energy-, cholinergic, glutamatergic, ERK/MAPK- and PKC-related pathways, respectively).** The agents were tested in the absence or presence of intracellular Ca^2+^ chelator BAPTA-AM (Bap, 10 nM) or energy metabolism inhibitor rotenone (Rot, 1 μM) in cultured SH-SY5Y cells. In the experiments testing the inhibitors, the cells were preincubated with Bap or Rot for 10 min before the addition of the agents. All values are means + SEM from at least five independent experiments. *p < 0.01; **p < 0.001, versus basal level. Nic, nicotine; Glu, glutamate; EGF, epidermal growth factor; PDBu, phorbol 12, 13-dibutylester. Methods for Western blotting to determine the sAPPα levels were as previously described (Chen and Fernandez, 2004).

In cultured SH-SY5Y cells we found that these five agents all robustly enhanced the release of sAPPα (secreted APP by α-secretase) to various degrees (**Figure 3**). But these effects were abolished by BAPTA-AM, an intracellular Ca^2+^ chelator and, at the same time, the effects were also blocked by rotenone, a respiratory chain complex 1 inhibitor (**Figure 3**). This suggests that α-secretase is both energy- and Ca^2+^-dependent.

**FIGURE 4:**
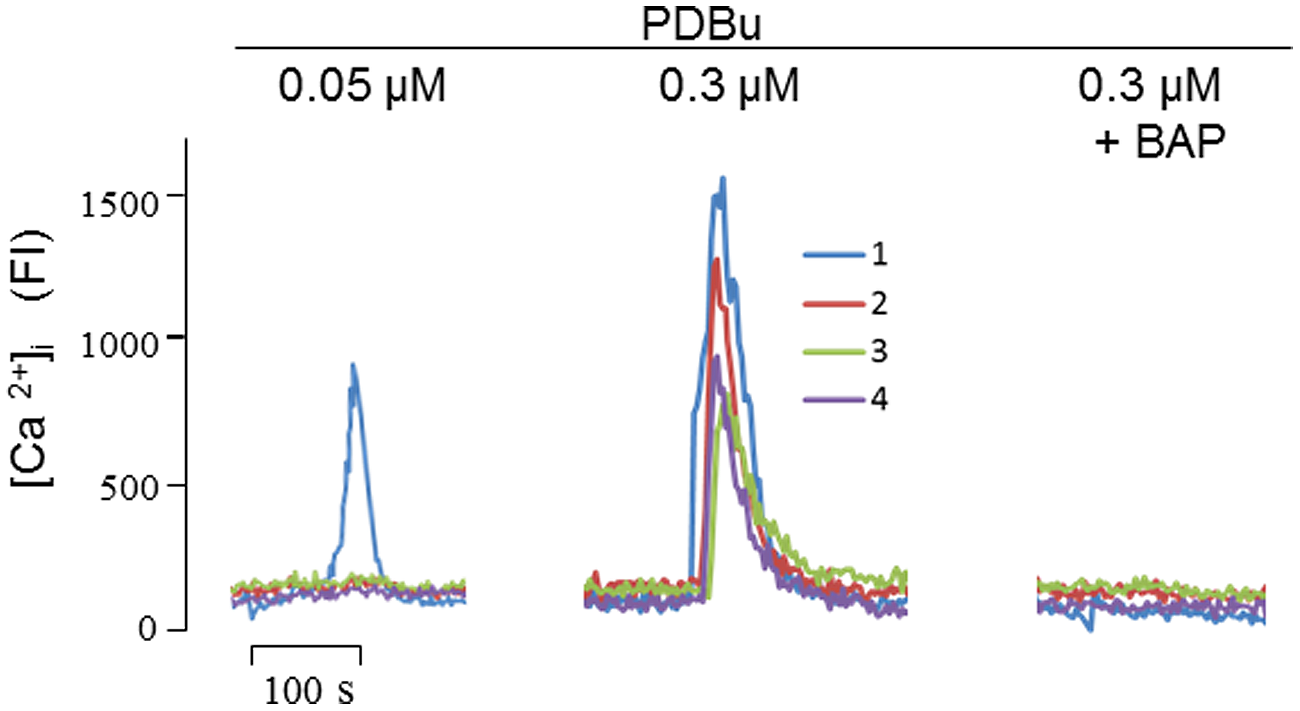
**Phorbol 12,13-dibutylester (PDBu) mobilized intracellular Ca^2+^ signals in cultured SH-SY5Y cells.** At the indicated concentrations, PDBu evoked fluorescent signals in sequential stimulations. In testing the inhibitory effect of Bap (10 nM), cells were preincubated with Bap for 10 min before the addition of PDBu. Four fluorescence traces were recorded corresponding to the four single cells focused by the laser scanning confocal microscope. FI, fluorescence intensity. Methods for intracellular Ca^2+^ imaging were described previously (Nguyen et al., 2013).

Furthermore, of the five agents we tested, the mechanism of PDBu needs to be further clarified, because the mode of actions of phorbol esters, the strongest stimulator for α-secretase when tested *in vitro*, has only been attributed to PKC activation today. Since PKC does not directly explain the *proteolytic cleavage* of APP, we speculated that phorbol esters may also activate α-secretase by mobilizing Ca^2+^, as PKC is a Ca^2+^-dependent enzyme (Kikkawa et al., 1982). To test this possibility, we carried out Ca^2+^ imaging and found that PDBu evoked Ca^2+^ transients in a concentration-dependent manner in cultured SH-SY5Y cells, an effect that was completely blocked by BAPTA-AM (**Figure 4**). Thus, among other actions, PDBu is also a potent Ca^2+^ activator.

## ENERGY-Ca^2+^ DEFICITS CAN ALSO EXPLAIN AMYLOID PLAQUES

The Ca^2+^-dependent feature of α-secretase has prompted us to suggest calpain as its candidate (Chen et al., 2000; Chen and Fernandez, 2004, 2005). However, question remains, because APP is a transmembrane protein, whereas calpain lacks a hydrophobic segment in its primary sequence, as we noted (Chen and Fernandez, 1998).

But, there are other proteases that are both Ca^2+^-dependent and transmembrane. In fact, furin or PC7, two members of the proprotein convertase family, have been reported to have both features and also are directly or indirectly involved in APP α-processing (Lopez-Perez et al., 1999; Hwang et al., 2006; Turpeinen et al., 2013). If such a protease (be it furin, PC7 or another Ca^2+^-dependent protease) is eventually proven to be α-secretase, then our model predicts that it should display biochemical properties similar to calpain, and that it must also be specific and exclusive in substrate recognition (otherwise Ca^2+^ signaling would be disrupted) and inactivated by aging. We thus believe that amyloid plaques are formed by a mechanism similar to the depositions of tau and other calpain substrates: Ca^2+^ signaling deficit (**Figure 2**).

The unsolved identity of α-secreatse thus should attract the attention of the research field with focused studies on it. Although it is no longer of key importance for sAD intervention (activating Ca^2+^ will activate it), we still believe that if this issue is resolved, then the three major diagnostic markers of sAD would be mechanistically understood in scientific terms. Unfortunately, however, most research attentions today are devoted instead to other competing models: metalloproteases (Buxbaum et al., 1998; Lammich et al., 1999) and β-/γ-secretases (Citron et al., 1997; Vassar et al., 1999). As discussed below, however, these other models, though popular, may face severe theoretical obstacles.

## THE THEORETICAL OBSTACLES FOR METALLOPROTEASES AS α-SECRETASE

(a) Metalloproteases require a metal as their catalytic center, not an activity regulator, so they belong to *unregulated* proteases (though they can be *affected* by the signal pathways), thus may not be the *most reasonable* candidates for α-secretase. (b) More importantly, if α-secretase is really an unregulated protease, then it would be difficult or impossible to modify its activity in the brain by common approaches (such as physical exercises). (c) None of the many other substrates of metalloproteases (Vingtdeux and Marambaud, 2012) is known to accumulate in aging (as our model predicted). (d) Metalloproteases initially appear to be α-secretase candidates by their “specific” cleavage on Aβ peptide *in vitro* (Buxbaum et al., 1998; Lammich et al., 1999), yet α-secretase is not sequence-specific but “distance-specific” (the distance of the cleavage site to the membrane; Maruyama et al., 1991; Sisodia, 1992). This key feature is imposed by the “double-anchorage” of both APP and α-secretase in the membrane (Chen, 1997), but difficult to mimic *in vitro*.

## CAN β- AND γ-SECRETASES BE RESPONSIBLE FOR Aβ GENESIS?

Over the years, however, the key importance of α-secretase – whatever its identity – has been severely overshadowed by a much more popular concept that Aβ is solely generated via an *abnormal* “amyloidogenic” pathway by “β- and γ-secretases.” So inhibiting them would cure AD without touching α-secretase (Selkoe, 2005). As the concept is so appealing, the β-/γ-secretases have been claimed to be “positively identified” and published in top-tier journals (Citron et al., 1997; Vassar et al., 1999), and thousands of studies have since followed.

We, however, stick to the biological laws first. It has come to our attention that such enthusiasms stem from the “disease” definition of sAD, so they may face hard questions. For example, if they are solely responsible for Aβ genesis, then β- and γ-secretases must be progressively *activated* by aging, that is, activated by *free radicals*, *energy crisis* and other age-related insults.

Can proteases be activated by such insults *in vivo*? This fundamental question now stands in front of us – regardless our subjective perceptions of sAD or philosophic faith – and demands a definitive answer. Further, if Aβ is generated by β-/γ-secretases, then how many other “β-/γ-secretases” would have to be assumed for tau and other deposited protein fragments? Are they all activated by aging?

Proteases and kinases (see above) are known to be activated by their increased quantity or functionality via up-regulated DNA replication, protein synthesis or signal transductions, etc. during physiological activities such as cell growth and memory formation. Now, if they are also “activated” by aging, an opposite process that diminishes those activities leading inevitably to a full stop in the end, then cogent mechanisms compatible with the established biological principles must be provided for why and how it can happen.

Perhaps, aging inactivates an intrinsic protease inhibitor, thereby activating the protease (Rao et al., 2008)? This scenario needs to explain in the first place why aging affects only one member of the pair. Yet, maybe proteases are activated by age-related *abnormalities* such as epigenetic changes, cell death, DNA damages, inflammations, immune defects or many other possible metabolic errors?

Again, the “disease” definition of sAD has allowed countless such “abnormalities,” replicas of pathogenic factors in discrete diseases, to be assumed and pursued. But it is a conceptually confused journey, because plaques and tangles, the common features of the normal aging brains, have to be logically explained by *normal* changes in aging, similar to cholesterol deposition.

## α-SECRETASE IS THE PRIMARY DETERMINANT FOR Aβ LEVELS

If age-related cholesterol deposition simply results from an inefficient lipid degradation – a *normal* event in aging, rather than from any “abnormal” lipid “depository pathway” or “activated” lipid “deposit-ases” – then plaques and tangles would also be formed by a similar and simple mechanism: insufficient proteolysis, at least at their initial phase.

It is well-known by now that most intact APP is α-processed in the young, leaving only a tiny amount of it to generate Aβ. Therefore, unless intact APP is somehow increased, there would be no chance whatsoever for Aβ to increase (likewise, unless lipid catabolism slows down, no cholesterol will deposit). Thus, α-secretase activity, which controls the levels of intact APP, would be the primary determinant or rate-limiting step for Aβ levels (i.e., other proteases can only play secondary roles; similar to tangle formation; **Figure 2B**). Yet, Ca^2+^-regulated α-secretase may not allow any other unregulated proteases to act more sensitively to “compete” with it for alternative APP cleavages (i.e., they have a chance only after α-secretase inactivation).

This concept, in fact, has been well-established by numerous *in vitro* and *in vivo* studies showing that activating α-secretase, alone, reduces Aβ; and conversely, inhibiting the enzyme, alone, increases Aβ [Chen, 1997; Chen and Fernandez, 2001b; and references therein]. Why is this knowledge being ignored in the pursuing of β-/γ-secreatses? Perhaps because it points to Aβ overproduction as a *natural* event in aging, contrasting with the “disease” definition.

The following considerations further suggest that “identification of β-/γ-secretases” is a problematic concept in inception: (i) most proteases are non-specific, so most peptides they produced in our body are unlikely to have *specific* and *singular* – thus identifiable – “secretases”; (ii) Aβ is no exception, as it exists *in vivo* not as Aβ1-40/42 only, but a mixture of many peptides with their *N*-termini starting from -4 -3, -2, +3 up to +9, and *C*-termini from 34, 38, 44, and up to 46 (Masters et al., 1985; Haass and Selkoe, 1993; Kim et al., 2001; Zhao et al., 2004). Such heterogeneous peptides are unlikely to be generated by two single proteases; (iii) indeed, several “β-site cleaving proteases” have been identified today, proving the *multiplicity* of “β-secretase.” If so, then what are the values for identifying one or a few of them? And (iv) if γ-secretase is a presenilin, then not only would it be “overactivated” by aging (no one has provided a reason for this assumption), but also by each of its near-200 gene mutations – a wild “gain of function” model that contrasts with a “loss of function” (memory) disease [note that our “mutations cause inefficient Ca^2+^ channeling” model better explains the roles of the mutations (Chen and Fernandez, 2001b; Chen et al., 2011b), which has been supported by an important recent study (Sun et al., 2014)].

We are aware that current models are corroborated by mountains of evidence, but they have not explained sAD. So it is necessary for us to keep an open mind. The supreme judge in science is *reason*, not only “evidence” (also note that not all published data can be called “evidence,” unless they offer a reasonable explanation for the disease).

## A BROAD NEW FRONTIER FOR sAD PREVENTION

Our model (**Figure 1**) points to energy and Ca^2+^ deficits as the two primary drug targets for early sAD intervention. As such, numerous energy-promoting and Ca^2+^-activating agents/practices would be useful. These include but are not limited to: physical exercise, glucose catabolism stimulators, energy-rich substances, growth factors, hormones, neurotransmitter receptor agonists, neurotrophic factors, and metabolic enhancers (Nguyen et al., 2013; Nguyen and Chen, 2014).

This would be a broad and new research frontier (compared to current dominant anti-amyloid and anti-calcium strategies). Notably, similar approaches have been successfully used to delay or prevent other senile disorders such as atherosclerosis and osteoporosis, and some of them have been shown to exhibit neuroprotective effects in sAD studies (Pettegrew et al., 2000; McDaniel et al., 2003; Chan and Shea, 2007; Ritchie et al., 2007; Fisher, 2012; Newhouse et al., 2012). Thus, it is reasonable to anticipate that early use of such drugs (or their more effective derivatives or their combinations), especially when enhanced by targeting many risk factors in late life via improved social supports, will significantly delay or even prevent sAD.

At the same time, our study warns that current enthusiastic clinical trials on anti-amyloid and anti-mutation drugs in high risky individuals for EOAD and FTD are not the most effective use of the invaluable resources, because they will not benefit, but only distract, our study focus on sAD, the most severe social threatening disease.

## CONCLUSION

Sporadic Alzheimer’s disease differs fundamentally from discrete diseases. This is the watershed where their study paradigms diverge but is also where commonsense and illusion collide.

In this work we further examined our “energy/Ca^2+^ deficits” model for its explanatory potential for several key sAD features in comparison with current models (e.g., Ca^2+^ overactivation, amyloid hypothesis, tau kinase activation, protein misfolding, and β-/γ-secretases). It appears that despite the controversies and paradoxes, our model can explain the sAD features better than other models.

Our model (**Figure 1**) also rests on several assumptions including a “ligand-receptor” relationship between calpain and substrates, “alternative breakdown” of calpain substrates, energy-/Ca^2+^-dual dependent α-secretase and its rate-limiting role in Aβ genesis. These assumptions may subject to modifications as new findings emerge in the future, but our starting point for reasoning – sAD initiates from natural aging – and the key intervention targets it suggests – energy and Ca^2+^ signaling deficits – may stand the test of time and reason.

Nevertheless, our model leaves a supreme question at large: if memory inefficiency, plaques and tangles all result from normal aging as we suggested, then what on earth can explain the devastating sAD only in some but not in other elderly (**Figure 1**; question mark)? This question should have been guiding the sAD research from the beginning, yet unfortunately, it has never been explicitly asked by NIA. As we discussed elsewhere (Chen and Fernandez, 2000; Chen et al., 2011b), this question can be hopelessly complicated to answer if sAD is as defined today, but it may be much simpler in concept if it is merely one of many *senile disorders*, as viewed by Alois Alzheimer and his colleagues by commonsense. NIA needs to return to commonsense in the perception of sAD.

## Conflict of Interest Statement

The authors declare that the research was conducted in the absence of any commercial or financial relationships that could be construed as a potential conflict of interest.
